# Procedural Pain Assessment in Infants Without Analgosedation: Comparison of Newborn Infant Parasympathetic Evaluation and Skin Conductance Activity - A Pilot Study

**DOI:** 10.3389/fped.2021.746504

**Published:** 2022-01-11

**Authors:** Wojciech Walas, Zenon P. Halaba, Tomasz Szczapa, Julita Latka-Grot, Iwona Maroszyńska, Ewelina Malinowska, Magdalena Rutkowska, Agata Kubiaczyk, Monika Wrońska, Michał Skrzypek, Julien De Jonckheere, Mickael Jean-Noel, Andrzej Piotrowski

**Affiliations:** ^1^Institute of Medical Sciences, University of Opole, Opole, Poland; ^2^Department of Pediatrics, Institute of Medical Sciences, University of Opole, Opole, Poland; ^3^Neonatal Biophysical Monitoring and Cardiopulmonary Therapies Research Unit, Department of Neonatology, Poznań University of Medical Sciences, Poznań, Poland; ^4^Neonatal Department, Children's Memorial Health Institute, Warszawa, Poland; ^5^Department of Intensive Care and Congenital Malformations of Newborns and Infants, Polish Mother's Memorial Hospital Research Institute, Łódz, Poland; ^6^Department of Neonatology, Institute of Mother and Child, Warszawa, Poland; ^7^Department of Anaesthesiology and Intensive Care, Children's Memorial Health Institute, Warszawa, Poland; ^8^Department of Biostatistics, School of Public Health, Medical University of Silesia, Bytom, Poland; ^9^INSERM CIC-IT 1403, Lille University Hospital, Lille, France; ^10^MDoloris Medical Systems, Loos, France

**Keywords:** pain, infant, SCA, NIPE, behavioral scales of pain

## Abstract

**Objective:** New technologies to measure pain responses, such as heart rate variability and skin conductance hold promise in the development of tools that can be reliable and quantifiable of detecting pain. The main objective of this study was to assess the capability of two monitors i.e., Newborn Infant Parasympathetic Evaluation (NIPE) and Skin Conductance Algesimeter for detecting procedural pain in non-anesthetized infants.

**Materials and Methods:** Thirty-three non-anesthetized infants were enrolled to the study. To detect pain caused by heel stick, NIPE, and Skin Conductance monitors and behavioral pain scales were used. Three minutes before and just after heel stick, pain was evaluated by behavioral scales, and simultaneously over the whole period by NIPE and SCA.

**Results:** A statistically significant decrease of NIPE Index and an increase of SCA values were found after the HS procedure. There were no statistically significant differences between the decrease in NIPEi values and the increase in PPS values between subgroups based on pain assessment by behavioral-scale scores.

**Conclusion:** Both NIPE and SCA can be useful for detection of procedural pain and may constitue an additional valuable tool for better handling of pain among patients treated in NICUs. More studies on larger groups of patients are needed.

## Introduction

Infants in neonatal intensive care units are exposed to numerous painful procedures each day as a part of routine care. Although there are some basic differences in the physiology of pain perception in infants, even preterm babies can perceive pain comparable to older children and adults. There is strong evidence that untreated or undertreated pain may impair their brain and cognitive function development (1–5). Repeated exposures to pain and stress during neonatal period have resulted in long term consequences including alterations in pain sensitivity and changes in brain structure and function (6). Pain assessment in neonates is challenging for caregivers and for this reason in some cases pain may not be treated effectively (7, 8). Self-reporting of pain is usually the standard for assessment of the presence and severity of pain, but it cannot be applicable in children below 3 years of age. In infants, acute pain may be assessed using pain assessment scales. Although, they present the gold standard in assessing severity of pain in non-verbal children, only some of them possess both strong validity criteria and are polyvalent. In addition, they require prolonged clinical observation, and pain scoring is intermittent which can lead to the overlooking of some painful episodes (9, 10). Hence there is a need to find for more objective and effective pain measurement tools. Over the last decades new non-invasive methods for the evaluation of pain or stress in infants and children have been applied, such as heart rate variability (HRV), skin conductance, pupillary reflex dilatation, and near-infrared spectroscopy (11–14). Among them Newborn Infant Parasympathetic Evaluation (NIPE) and Skin Conductance Activity (SCA) are becoming popular. They both are proposed to be objective, reliable tools for neonatal pain, stress and discomfort evaluation but only few infant studies have yet validated them. The NIPE method is based on evaluating the parasympathetic nervous system tone changes and SCA uses changes in sympathetic nervous system tone.

The main objective of this prospective, observational study was to assess the capability of these two devices i.e., NIPE monitor and Skin Conductance Algesimeter for detecting procedural pain in non-anesthetized infants. The second objective was to evaluate the influence of gestational age, type of breathing (spontaneous or non-invasive ventilatory support), birth weight and weight at the time of evaluation on obtained NIPE and SCA values.

To the best of our knowledge, this is the first study that compares NIPE and SCA in the clinical setting of NICUs.

## Materials and Methods

### Patients

This multicenter prospective observational pilot study was performed at six distinct N/PICUs in Poland between 15 October and 31 December 2018. For this pilot study, 33 newborns and infants admitted to N/PICUs were enrolled. They all were breathing spontaneously or receiving only non-invasive respiratory support and did not require analgesia and/or sedation. Inclusion criteria were gestational age >26 weeks and postnatal age <3 months. Exclusion criteria included bradycardia <80/min., tachycardia >200/min., except for transient episode (<15 s) of sinus bradycardia or tachycardia, cardiac rhythm different from sinus rhythm, the use of catecholamines or other drugs influencing the autonomous nervous system (e.g., beta blockers) during a period of seven days before the study, intraventricular hemorrhage - grade IV, inborn brain malformations, severe birth asphyxia treated with therapeutic hypothermia, neuromuscular disorders, and seizures in the seven days preceding the study. All guardians were given information forms, gave their approval for the study, and written informed consent was obtained from the parents of all participating patients. This was a prospective, observational study which did not require any changes regarding the standard treatment of patients included. The acceptance of the local Ethical Committee was granted (270: 11.10.2018).

The characteristic of the studied group is presented in Table 1.

**Table 1:** Characteristics of the studied group.

**Overall characteristics** **(*n* = 29)**	Mean ± SD^*^ Median (1^**st**^-3^**rd**^ quartile)^†^ *n (%)*
Gestational age (weeks)	35 (31–39)^†^
Birthweight (g)	2218 (1310–3215)^†^
Female	14 (48)
Male	15 (52)
**Characteristics at the time of the events** **(*n* = 36)**	
Age (days)	14 (4–39)^†^
Postmenstrual age (weeks)	38 ± 4^*^
Weight (g)	2622 ± 938^*^
Spontaneously breathing	20 (56)
Non-invasive ventilation	16 (44)

* *Normal distribution*,

† *non-normal distribution, according to the Shapiro-Wilk test*.

### Methods

To prevent pain oral sucrose was used as a non-pharmacological measure before heel lancing in all patients. Fifteen minutes before a due standard heel stick (HS) for capillary blood sampling, continuous monitoring of HRV by means of NIPE monitor (Mdoloris Medical Systems, Loos, France) and continuous SC monitoring by means of Skin Conductance Algesimeter (SCA MedStorm Innovation, Norway) were started. Patients stayed calm over the 15-min period before HS. A comprehensive description of the NIPE methodology has been published by De Jonckheere et al. (13). The NIPE monitor displays two values of the NIPE index: the NIPEm is computed as a mean value over 20 min, whereas the instantaneous NIPEi provides information regarding short-term HRV-analysis, showing the result of a 64-s moving window, with an update frequency of 1 s. This monitor presents values from 0 to 100 points; the stronger the pain, the lower the result displayed. In this study, we used only the NIPEi, as we intended to examine acute changes after a noxious stimulus.

Description of the SC methodology has been published by Storm (15, 16). Our results are presented as Peaks per Second (PPS), increasing from 0, in response to pain stimuli. PPS is the main index used for assessing SC measurements, which is the best validated SC index for pain scoring in infants. In practice, the time of blood collection is different from patient to patient, and the reaction of the studied monitors presented as changes of PPS and NIPEi also develops differently, due to algorithms incorporated in the devices (13, 15). This is the reason for including the level of NIPEi and PPS 1 min before painful stimulus (NIPEi-1, PPS-1), and the minimal value of NIPEi (NIPEi min.), as well as maximal value of PPS (PPS max.), during the 3-min period after HS. Experienced pain was assessed using behavioral scales 3 min before, and at the time of performing HS. For premature infants up to 36 weeks postmenstrual age (PMA) the Premature Infant Pain Profile (PIPP) was used, while for infants over 36 PMA, up to 2 months of age, the Neonatal Infant Pain Scale (NIPS) was employed. For older babies the Face, Legs, Activity, Cry, Consolability scale (FLACC) was used. PIPP is a 7-indicator composite measure. The score ranges from 0 to 21, with the higher score indicating more pain (17, 18). NIPS is a six-indicator composite measure. Results are obtained by summing up the scores for the six indicators, where 0 indicates no pain, and a score >3 indicates pain, with a maximum score of 7 (19). FLACC includes five indicators. The scale is scored in a range of 0–10, with 0 representing no pain. It is used to evaluate pain in pre-verbal children from 2 months to 7 years (20). Following the principles of treating pain in newborns, we divided the studied population into two subgroups depending on the pain scale scores: no/mild/moderate pain (PIPP 0 – 12, NIPS 0 – 4, FLACC 0 – 6), and severe pain (PIPP > 12, NIPS > 4, FLACC > 6). Trained observers (neonatologists) scoring the sensation of pain did not have access to the NIPE index and PPS values.

### Statistical Analysis

The statistical analysis was performed using SAS software version 9.4 (SAS Institute Inc., Cary, North Carolina, USA) and R version 3.5.1 (The R Foundation for Statistical Computing). Normality assumption was checked using the Shapiro-Wilk test. In subgroups, depending on the assessment of pain in the behavioral scale, comparison of NIPEi values 1 min before HS was performed using the Mann-Whitney U test. Comparisons of NIPEi values 1 min before, as well as the lowest values within 3 min after HS, the comparison of PPS values 1 min before, and the highest values 3 min after HS, was performed for the whole group and for subgroups, using a Wilcoxon signed rank test. For multiple comparisons, the Holm-Bonferroni method was used. We considered a *p* < 0.05 as significant. The influence on the decrease of NIPEi and elevation of PPS by the gestational age, postnatal age, type of breathing (spontaneous or non-invasive ventilatory support), birth weight and weight on the study day, as well as that of postmenstrual age, was evaluated using multivariable linear regression analysis.

## Results

**Figure 1:**
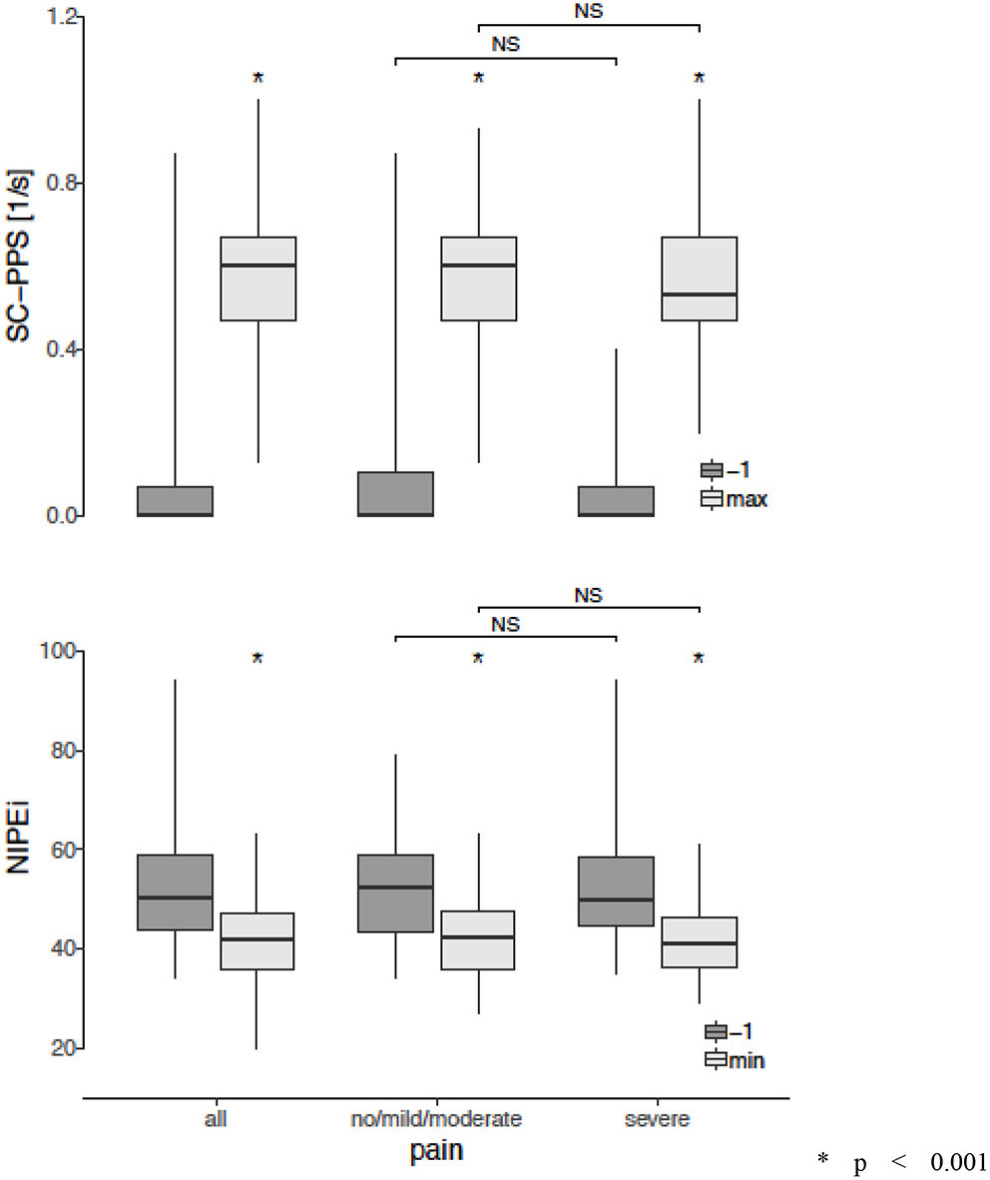
Changes in the Newborn Infant Parasympathetic Evaluation Index (NIPEi) and in the Skin Conductance (SC - PPS) after a painful stimulus in the whole group and in the subgroups according to assessment of pain in behavioral scales. *p < 0.001 (Wilcoxon signed-rank test, with Holm-Bonferroni correction for multiple comparisons in case of no/mild/moderate and sever subgroups) NS, non-significant (Mann-Whitney U test with Holm-Bonferroni correction for multiple comparisons).

In four patients enrolled to the study (12%), the NIPE and SCA signals (PPS) were distorted by artifacts due to movements and could not be interpreted. In the final analysis, 29 babies were included, in whom 36 HS procedures were analyzed. One minute before HS, no pain was detected in any patients according to the behavioral-scale scoring. Table 2 shows the NIPEi and PPS values 1 min before, as well as the lowest NIPEi levels and the highest PPS levels within 3 min after HS, in all patients and in subgroups based on pain assessment by behavioral-scale scores. Changes of NIPEi and PPS values in the whole group, as well as in the subgroups, are shown in Figure 1. There were no statistically significant differences between NIPEi, or between PPS values, in the subgroups 1 min before HS. We have observed a statistically significant decrease in NIPEi values and a statistically significant increase in PPS values after a painful stimulus, both in the whole group and in the subgroups, but there were no statistically significant differences between the decrease in NIPEi values and the increase in PPS values between subgroups. In multivariable linear regression analysis, there was no influence of gestational age, postnatal age, type of breathing (spontaneous or supported), birth weight and weight on the study day or postmenstrual age on the decrease of NIPEi and elevation of PPS after the HS.

**Table 2:** NIPEi and PPS values 1 min before and minimum NIPEi and maximum SC values within 3 min after HS in the whole group and in subgroups based on the pain assessment by behavioral-scale scores.

	**NIPEi-1**	**NIPEi min**.	**PPS-1**	**PPS max**.
Whole group *n* = 36	50.5 (44.0–59.0)	42.0 (35.5–47.0)	0.00 (0.00–0.14)	0.60 (0.47–0.73)
	p < 0.001^*^		p < 0.001^*^	
No/mild/moderate pain group *n* = 16	52.5 (43.0–59.0)	42.5 (35.5–48.0)	0.00 (0.00–0.14)	0.64 (0.47–0.74)
	*p* = 0.00^†^		*p* < 0.001^†^	
Severe pain group *n* = 20	50.0 (44.5–59.0)	41.0 (35.5–46.5)	0.00 (0.00–0.07)	0.57 (0.47–0.73)
	*p* < 0.001^†^		*p* < 0.001^†^	

*The results are presented as median (the lower quartile - the upper quartile)*.

* *Wilcoxon signed rank test*.

† *Wilcoxon signed rank test with Holm-Bonferroni correction for multiple comparisons*.

## Discussion

The assessment of acute pain should be an essential component of infants' care in NICUs and should lead to improved pain control. However, assessment of pain is complicated and mainly based on behavioral scales which are still the gold standard in evaluating severity of pain in this age group of patients, they are not free from bias and have some limitations. New technologies to measure pain responses, such as heart rate variability and skin conductance, seem to be promising in the development of tools that can be reliable, and capable of detecting pain. The main objective of our study was to evaluate the ability of the Newborn Infant Parasympathetic Evaluation monitor and Skin Conductance Algesimeter to detect procedural pain sensation in infants. To our knowledge, this is one of the first studies evaluating concurrently the capability of these two methods for assessing procedural pain in non-anesthetized infants. In our study we have observed the statistically significant decrease of NIPEi values and increase of PPS values after a painful procedure. It may indicate the ability of both devices to detect pain in infants. Similar results regarding SCA have been obtained by Munsters et al. They have shown that a stress response to heel stick can be detected with skin conductance measurements from 22 weeks GA (21). Also, Tristão et al. have demonstrated the usefulness of SCA in detecting pain after a daily routine prick (22). In regard to the NIPE method Zhang et al. in their study on 55 children aged 1 month to 2 years undergoing elective surgery, have noticed a statistically significant decrease in the NIPE value after endotracheal intubation and skin incision (23). Similar results were also obtained by Walas et al. in their pilot study on 36 non-anesthetized infants admitted to neonatal/pediatric ICUs. They have observed the statistically significant decrease in the NIPE value within 3 min after the painful stimulus, and the area under the receiver operating characteristic (ROC) curve computed between the NIPE value at rest and the minimum NIPE value over 3 min after a painful stimulus was 0.767 [95% CI 0.666; 0.868] for the whole studied group (24). In another way, Genderas et al. assessing pain in hospitalized premature infants did not find a significant correlation between NIPE index and Premature Infant Pain Profile scale, however they have shown that as the NIPE index as SCR, both had high sensitivity and high negative predictive values to predict PIPP-R > 10, especially for skin-breaking painful procedures (25). In our study the decrease in NIPEi values and the increase in PPS values were not statistically different between the subgroups of different pain intensity formed after behavioral assessment. It may be explained by too small number of the studied subgroups. However, the ability of NIPE and SCA monitors to evaluate the pain intensity in infants is equivocal and some authors have observed considerable variability of responses to painful and other unpleasant, stressful stimuli (26, 27). Pereira-da-Silva et al., De Jesus et al., and Eriksson et al. have compared the results of SC measurements with behavioral scale assessment in response to HS in neonates and observed no correlation, although significant changes after the painful stimuli were noted (12, 28, 29). Storm et al. have shown that SC changes after a pain stimulus in premature neonates did not correlate with a four-step behavioral scale (15). On the contrary, Tristão and colleagues have observed a correlation between a rise in SC values and assessment done in a modified COMFORT scale, in response to HS in neonates (22). Also, Dalal et al. have shown a similar correlation between SC and NFCS scale in 6–12 months old infants after surgery (30). Just as the SC results, there are conflicting results with regard to the correlation between HRV and assessment based on behavioral scales. Cremillieux et al. assessed responses in premature neonates and have presented results similar to ours: no correlation between changes of NIPEi and those measured using DAN and PIPP-R scales (31). Valencia-Ramos et al. have found no NIPEi and COMFORT scale correlation in neonates during the nebulisation procedure; and nor did Okur et al., who used NIPEi and PIPP scale during surfactant administration in premature infants (32, 33). On the contrary, Weissman et al. have studied pain manifestation after HS using the Neonatal Facial Coding System and found a correlation with spectral analysis of heart rate variability (34). Also, Faye et al. and Buyuktiryaki et al. have noticed a correlation between different pain scales in the post-operative period, in term, as well as preterm, neonates (35, 36). To date, there are only two studies analyzing simultaneously SC and HRV changes after a painful stimulus. Sabourdin et al. have found that HRV analysis was superior to haemodynamic changes and SC measurement in children aged 3–15 years undergoing general anesthesia. In that study, the ANI device, working on the same principle as NIPE, was used, although only 12 patients and 11 traces were analyzed. Also, a different parameter of skin conductance from that suggested by the manufacturer was incorporated into the analysis (37). De Jesus and colleagues have analyzed the changes of oxygen saturation, heart rate variability and SC in term infants after HS and found significant reactions to painful procedures although the HRV analysis was performed by a method different than that used by the NIPE monitor (38). It is worth emphasizing that in our study we could observe that gestational age, postnatal age, type of breathing, birth weight and weight at the time of assessment do not affect the decrease in NIPEi and increase in PPS in response to the painful stimulus. This might suggest the ability of both methods in detecting pain in various patients and various clinical situations, but this would require further studies on a larger patient group. Our study has some limitations. The study group was small, and consisted only of newborns breathing spontaneously, without analgosedation. Furthermore, they were of various degrees of prematurity and chronological age.

## Conclusion

Both NIPE and SCA are useful for detection of procedural pain and may constitute an important tool for better handling of pain among patients treated in NICUs. Further studies are needed to assess the ability of these devices to measure the intensity of pain. In our study, such factors as sex, gestational age, postmenstrual and postnatal age, birth weight and weight at study, type of breathing (spontaneous or non-invasive ventilatory support) had no influence on the ability to detect pain after a procedural intervention.

## Data Availability Statement

The raw data supporting the conclusions of this article will be made available by the authors, without undue reservation.

## Ethics Statement

The studies involving human participants were reviewed and approved by Medical Ethics Committee of Regional Medical Chamber in Opole, Poland. Written informed consent to participate in this study was provided by the participants' legal guardian/next of kin.

## Author Contributions

WW, ZH, TS, and JL-G was involved in the study conception and design, requested the data sets, and drafted the manuscript. IM, EM, MR, AK, and MW requested the data sets and drafted the manuscript. MS, JD, and MJ-N was involved in the study conception and design and provided statistical support. AP was involved in the study conception and design and critically reviewed the draft manuscript. All authors approved the final manuscript as submitted and agree to be accountable for all aspects of the work.

## Conflict of Interest

MJ-N was employed by MDoloris Medical Systems. The remaining authors declare that the research was conducted in the absence of any commercial or financial relationships that could be construed as a potential conflict of interest.

## Publisher's Note

All claims expressed in this article are solely those of the authors and do not necessarily represent those of their affiliated organizations, or those of the publisher, the editors and the reviewers. Any product that may be evaluated in this article, or claim that may be made by its manufacturer, is not guaranteed or endorsed by the publisher.
